# Elevated Serum Triglyceride and Retinol-Binding Protein 4 Levels Associated with Fructose-Sweetened Beverages in Adolescents

**DOI:** 10.1371/journal.pone.0082004

**Published:** 2014-01-27

**Authors:** Te-Fu Chan, Wei-Ting Lin, Yi-Ling Chen, Hsiao-Ling Huang, Wei-Zeng Yang, Chun-Ying Lee, Meng-Hsueh Chen, Tsu-Nai Wang, Meng-Chuan Huang, Yu-Wen Chiu, Chun-Chi Huang, Sharon Tsai, Chih-Lung Lin, Chien-Hung Lee

**Affiliations:** 1 Department of Obstetrics and Gynecology, Graduate Institute of Medicine, College of Medicine, Kaohsiung Medical University, Kaohsiung, Taiwan; 2 Department of Obstetrics and Gynecology, Kaohsiung Medical University Hospital, Kaohsiung Medical University, Kaohsiung, Taiwan; 3 Institute of Environmental and Occupational Health Sciences, National Yang Ming University, Taipei, Taiwan; 4 Department of Nuclear Medicine, Kaohsiung Medical University Hospital, Kaohsiung Medical University, Kaohsiung, Taiwan; 5 Department of Oral Hygiene, College of Dental Medicine, Kaohsiung Medical University, Kaohsiung, Taiwan; 6 Department of Public Health, College of Health Sciences, Kaohsiung Medical University, Kaohsiung, Taiwan; 7 Department of Family Medicine, Kaohsiung Medical University Hospital, Kaohsiung, Taiwan; 8 Department of Public Health and Environmental Medicine, Kaohsiung Medical University, Kaohsiung, Taiwan; 9 Department of Nutrition and Dietetics, Kaohsiung Medical University Hospital, Kaohsiung, Taiwan; 10 Health Policy and Systems Management Program, Health Sciences Center, School of Public Health, Louisiana State University, New Orleans, Louisiana, United States of America; 11 Department of Laboratory Medicine, Kaohsiung Municipal Hsiao-Kang Hospital, Kaohsiung, Taiwan; 12 Department of Surgery, Kaohsiung Medical University Hospital, Kaohsiung Medical University, Kaohsiung, Taiwan; Paris Institute of Technology for Life, Food and Environmental Sciences, France

## Abstract

**Background:**

The metabolic effect of fructose in sugar-sweetened beverages (SSB) has been linked to de novo lipogenesis and uric acid (UA) production.

**Objectives:**

This study investigated the biological effects of SSB consumption on serum lipid profiles and retinol-binding protein 4 (RBP4) among Taiwanese adolescents.

**Methods:**

We evaluated the anthropometric parameters and biochemical outcomes of 200 representative adolescents (98 boys and 102 girls) who were randomly selected from a large-scale cross-sectional study. Data were analyzed using multiple regression models adjusted for covariates.

**Results:**

Increased SSB consumption was associated with increased waist and hip circumferences, body mass index (BMI) values and serum UA, triglyceride (TG) and RBP4 levels. Adolescents who consumed >500 ml/day of beverages half-to-heavily sweetened with high-fructose corn syrup (HFCS) exhibited TG and RBP4 levels 22.7 mg/dl and 13.92 ng/ml higher than non-drinkers, respectively. HFCS drinkers with hyperuricemia had higher TG levels than HFCS drinkers with normal UA levels (98.6 vs. 81.6 mg/dl). The intake of HFCS-rich SSBs and high value of BMI (≥24) interactively reinforced RBP4 levels among overweight/obese adolescents. Circulating RBP4 levels were significantly correlated with weight-related outcomes and TG and UA concentration among HFCS drinkers (*r* = 0.253 to 0.404), but not among non-drinkers.

**Conclusions:**

High-quantity HFCS-rich beverage consumption is associated with higher TG and RBP4 levels. Hyperuricemia is likely to intensify the influence of HFCS-rich SSB intake on elevated TG levels, and in overweight and obese adolescents, high BMI may modify the action of fructose on higher circulating levels of RBP4.

## Introduction

Retinol-binding protein 4 (RBP4)—the single specific transporter of vitamin A in blood—is a lipocalin-family protein secreted by mature adipocytes and activated macrophages in adipose tissue [1], [2]. Biochemical investigations of lean, obese and diabetic participants have demonstrated that the concentration of serum RBP4 increases before frank diabetes develops [3]. In insulin-resistant condition, the expression of the insulin-stimulated glucose-transporter 4 (GLUT4) is selectively reduced in adipocytes [4]. Animal studies have shown that serum RBP4 levels are elevated in the adipose tissue of mice with genetic knockout of adipose-specific GLUT4 [5]. In participants with various clinical presentations, serum RBP4 has been positively correlated with insulin resistance and is an important adipokine associated with type 2 diabetes and cardiovascular complications [3], [6]–[9]. Recent studies have suggested that RBP4 may produce a molecular connection between obesity and insulin resistance [10].

Epidemiological data from large cross-sectional investigations and well-powered prospective studies have revealed that the upsurge in obese children and adults is associated with increasing levels of sugar-sweetened beverage (SSB) intake [11], [12]. Sugar consumption from beverages in several populations contributes more than 15% to dietary energy [13]–[15]. The World Health Organization suggests that this level should not exceed 10% [16]. Although the American Heart Association recommends that added sugar consumption should not exceed 80 kcal/day for women and 150 kcal/day for men [17], recent reports have determined that daily caloric intake from SSBs in US children from 1999 to 2004 was 242 kcal/day for girls and 357 kcal/day for boys [18]. In overweight and obese adults, the metabolic effects of fructose in SSBs have been shown to increase the accumulation of visceral adipose tissue and induce hepatic de novo lipogenesis (DNL) and high lipid profile levels [19], [20]. Compared with glucose-sweetened beverages, consuming fructose-sweetened beverages has been linked to a higher circulating level of uric acid, RBP4 and gamma-glutamyl transferase activity in adults with body mass index (BMI) values of 25 to 35 kg/m^2^ [21]. However, it is unclear whether the metabolic effect of SSBs extends to adolescents.

Between 1980 and 1996, the prevalence of adolescent obesity in Taiwan has increased by 25.8% in boys and 27.7% in girls [22]. A recent study found that 87.7% of adolescents in Taiwan ingest at least one SSB serving per week, and that consuming high fructose-containing beverages has a significant effect on increased levels of BMI and serum uric acid (UA) [23]. The national Nutrition and Health Survey in Taiwan found that 59.8% and 30.3% of male and female teenagers aged between 13 and 18 years had hyperuricemia [24]. These findings emphasize the importance of investigating the obesity and hyperuricemia-related metabolic effects of SSB intake in Taiwanese adolescents.

This study investigates the biological effect of SSB intake on serum lipid profiles and RBP4. It also examines the interactive effects of fructose-rich beverage consumption, excessive body weight and hyperuricemia on clinical outcomes among Taiwanese adolescents.

## Methodology

### Participants

To evaluate SSB-associated metabolic effects, 200 adolescents (98 boys and 102 girls) aged between 12 and 16 years were randomly selected from the SSB consumption group of a large-scale representative study conducted to monitor Multilevel Risk Profiles for Adolescent Metabolic Syndrome (mRP-aMS) in urban, suburban and rural areas of Southern Taiwan [23]. The mRP-aMS study included 3784 randomly selected students from 36 junior high schools. A detailed description of the study can be found in Lin et al [23]. Because acute illness, injury and liver and renal failure may affect serum RBP4 [9], adolescents with these conditions were excluded from the study. In this investigation, participants were randomly selected from the non-consumption (n = 40), 1-to-350-ml/day consumption (n = 40), 351-to-500-ml/day consumption (n = 40), 501-to-750-ml/day consumption (n = 40) and >750-ml/day consumption (n = 40) of SSBs groups. Because the effects of consuming 1 to 350 ml/day and 351 to 500 ml/day of SSBs were similar, we combined the two groups in data analyses. The Institutional Review Board of Kaohsiung Medical University reviewed and approved the research, which was conducted in accordance with their guidelines. Written informed consent was obtained from all participants and guardians/caretakers on the behalf of the participants before their inclusion in the study.

### Data Collection

Class teachers and two research staff members used a developed structured questionnaire to collect research data, including demographic factors, personal disease history, lifestyle behaviors, dietary intake, physical activity, and smoking, alcohol consumption and betel-quid chewing status. Daily dietary intake of 23 food groups during the previous month was assessed for each participant using the semi-quantitative food-frequency questionnaire. The Taiwanese Food and Nutrient Databank, created by the national Food and Drug Administration, was used to estimate daily total calories according to the food intake information [25]. Data on physical activity in a representative week were also collected and converted into metabolic equivalent task (MET) minutes per week by multiplying the MET level of activity by the minutes of activity per day multiplied by the number of days per week [23]. The total MET-minutes per week were estimated by adding the calculated values of all activities. Adolescents were grouped into three categories based on their total physical activity tercile. Tobacco and alcohol use were defined as having one or more cigarettes and alcoholic drinks in the previous month, respectively.

The food-frequency questionnaire was used to obtain information on SSB intake, including soft drinks, fruit drinks and sweetened tea. SSB non-drinkers were defined as adolescents who consumed no more than one serving of any SSB per week. Total SSB consumption per day was calculated and classified according to a typical serving size in Taiwan. Adolescents consume two main types of SSBs. The first are hand-shaken sugar-containing drinks (HSDs), which are instant SSBs sold in hand-shaken beverage shops. Commonly consumed HSDs include green tea, bubble black tea, pearl milk tea and boba milk tea. Because this type of SSB is only sweetened with high-fructose corn syrup (HFCS; mainly HFCS-55), they are referred to as HFCS-sweetened beverages. Bottled sugar-containing drinks (BSDs) are the second type of SSB. Manufacturers decide how much sugar is added to BSDs. Granulated sugar (sucrose) is the primary caloric BSD sweetener, however certain BSDs use HFCS or a mixture of HFCS and granulated sugar. This type of SSB is referred to as a mixed sugar-sweetened BSD. In Taiwanese hand-shaken beverage shops, customers can select the sweetness of their HSD, thus HSDs are further categorized as slightly, half and heavily HFCS-sweetened drinks. A 750-ml slightly, half and heavily sweetened green tea and bubble black tea contain approximately 17, 25 and 50 ml (22, 34 and 68 g) HFCS, respectively.

### Anthropometric Measurement

Trained and certified researchers conducted anthropometric measurements using a standardized procedure after questionnaire data collection. Parameters measured included height, weight, waist and hip circumference, body fat and blood pressure. Body fat percentage was identified using a body impedance technique (BF-800, Tanita Corp, Tokyo, Japan) and participant BMI was calculated as weight divided by height squared (kg/m^2^). BMI values were used to determine overweight (≥24 to <27 kg/m^2^) and obese (≥27 kg/m^2^) participants according to the criteria defined by the Department of Health, Executive Yuan of Taiwan [26].

### Clinical Measurement

Blood samples were collected from adolescents after three weeks of data collection and anthropometric measurements. Research specimens were obtained in the morning by venipuncture after a 10-hour overnight fast. Lipid profile concentrations, including total cholesterol, triglycerides (TGs), high-density lipoprotein cholesterol and low-density lipoprotein cholesterol, were enzymatically measured by a chemistry auto-analyzer using commercially available reagents (TBA-c16000 automatic analyzer, Toshiba, Tokyo, Japan) [27]. The serum UA level was analyzed using an enzymatic colorimetric assay that uses the enzyme uricase to oxidize UA and produce allantoin and hydrogen peroxide [28]. Adolescent boys and girls with UA levels ≥7 mg/dl and ≥6 mg/dl, respectively, were defined as hyperuricemia [29]. The serum RBP4 concentration was quantitatively determined using an ELISA kit K6110 according to manufacturer standards (Immundiagnostik AG, Bensheim, Germany). RBP4 levels in the samples were quantified by referring their optical density at 450 nm to a Lot-dependent master calibration curve and using a calibrator with each test. The intra- and inter-assay coefficients of variation were 5% and 9.8%, respectively.

### Statistical Analysis

Multiple linear regression models were used to evaluate the association of SSB consumption with anthropometric and clinical parameters. Multivariate-adjusted differences (that is, partial regression coefficients) were used to assess the effect of SSB consumption level on continuous outcomes. In these models, multivariate-adjusted means reflect the estimated predictions when covariates were set as mean values. A basic regression model including age, gender and study area was used to appraise potential confounders. Variables that altered the effect of interest by >10% or that had been established as confounders by previous studies were regarded as confounding factors [30], [31]. Pearson's correlation tests were used to examine the possible linear association of RBP4 with anthropometric and clinical parameters among adolescents who consumed different types of SSBs. Cross-product terms of explanatory variables were introduced into multiple regression models to evaluate the interaction effects of SSB intake, overweight/obesity and hyperuricemia on serum TG and RBP4 levels.

## Results

Table 1 shows participant background data, including demographic, dietary and physical factors, by SSB consumption group. Participants who ingested more SSBs tend to be boys, consume more calories and are more likely to drink alcohol. In adolescents who consumed >750 ml/day of SSBs, 67.5% drank half or heavily HFCS-sweetened HSDs, and 50% of adolescents who consumed <500 ml/day drank mixed sugar-sweetened BSDs.

**Table 1:** Demographic, dietary and physical factors associated with consumption of sugar-sweetened beverage in adolescents.

Factors	Sugar-sweetened beverage intake (ml/day)				*P* a
	Non-intake	1–500	501–750	>750	
**Study sample (no.)**	**40**	**80**	**40**	**40**	
**Demographic factor**					
Age (years)^b^	13.4±1.0	13.5±1.0	13.5±1.2	13.6±1.2	0.803
Boy	25.0%	51.3%	47.5%	70.0%	0.001*
Study area					
Urban	65.0%	47.5%	47.5%	57.5%	0.625
Suburban	15.0%	26.3%	25.0%	22.5%	
Rural	20.0%	26.3%	27.5%	20.0%	
**Dietary and physical factor**					
Total calories (kcal/day)^b^	1875.5±501.5	1977.1±591.0	2169.2±590.5	2307.6±1050.4	0.020*
Physical activity (MET-min./week)					
<952.5	35.0%	23.8%	27.5%	20.0%	0.479
952.5–2140.4	45.0%	41.3%	40.0%	37.5%	
≥2140.5	20.0%	35.0%	32.5%	42.5%	
Alcohol drinking	0.0%	15.0%	20.0%	15.0%	0.043*
Cigarette smoking	2.5%	2.5%	5.0%	10.0%	0.266
**Type of SSB**					0.017*
HFCS HSD (sweetened)					
Slightly		15.0%	20.0%	12.5%	
Half		18.8%	22.5%	30.0%	
Heavily		16.3%	30.0%	37.5%	
Mixed-sugar BSD		50.0%	27.5%	20.0%	

**Abbreviations**: HFCS, high-fructose corn syrup; HSD, hand-shaken sugar-containing drink; BSD, bottled sugar-containing drink;

* , *P*<0.05.

a *P* for mean or proportion difference across the user groups of sugar-sweetened beverages.

b Data are presented as mean±SD.

Table 2 shows the multivariate-adjusted differences in anthropometric and metabolic parameters associated with SSB consumption. High-amount SSB intake (>750 ml/day) was related to high weight and waist and hip circumferences (*P*<0.05), and increased SSB intake was linked to increased BMI values; serum UA, TG and RBP4 levels; overweight/obesity percentages and hyperuricemia (*P* for trend <0.05). Adolescents who consumed >750 ml/day of SSBs had UA, TG and RPB4 concentrations 1.28 mg/dl, 30.28 mg/dl and 15.87 ng/ml more than non-drinkers, respectively. All SSB drinkers had higher mean levels of RBP4 (11.59 to 15.87 ng/ml increases in mean).

**Table 2:** Multivariate-adjusted differences^a^ in anthropometric and metabolic parameters associated with sugar-sweetened beverage consumption in adolescents.

	Sugar-sweetened beverage intake (ml/day)								
Factors	Non-intake		1–500		501–750		>750		*P*_trend_
	aMean^b^	SE	Diff.	SE	Diff.	SE	Diff.	SE	
**Anthropometric parameter**									
Weight (kg)	53.95	1.94	−0.98	2.37	−1.98	2.72	5.54*	2.80	0.061
Waist circumference (cm)	70.72	1.61	−0.45	1.97	−0.51	2.26	5.21*	2.32	0.025
Hip circumference (cm)	90.10	1.47	−0.90	1.79	−1.12	2.06	6.17*	2.12	0.004
Body fat (%)	24.30	1.55	−2.70	1.89	−3.00	2.17	2.61	2.23	0.194
Body mass index (kg/m^2^)	21.01	0.67	−0.19	0.82	−0.44	0.94	2.34*	0.97	0.020
Overweight/obesity (%)^c^	13.31	6.60	4.84	8.04	−0.67	9.23	24.44*	9.50	0.025
**Metabolic parameter**									
Uric acid (mg/dl)	5.43	0.22	0.42	0.26	0.45	0.30	1.28*	0.31	<0.001
Hyperuricemia (%)	16.47	7.29	11.40	8.89	12.32	10.20	35.01*	10.51	0.002
Total cholesterol (mg/dl)	162.14	6.11	−4.81	7.45	2.43	8.55	−2.18	8.80	0.867
Triglyceride (mg/dl)	68.25	3.56	7.75	8.25	5.82	9.48	30.28*	9.76	0.004
HDL-C (mg/dl)	57.22	1.94	0.33	2.37	0.66	2.72	−4.72	2.80	0.098
LDL-C (mg/dl)	89.72	5.32	−4.89	6.48	2.98	7.44	2.79	7.66	0.381
RBP4 (ng/ml)	18.99	1.20	11.59*	1.46	13.84*	1.68	15.87*	1.73	<0.001

**Abbreviations**: aDiff., adjusted difference; SE, standard error; HDL-C, high-density lipoprotein cholesterol; LDL-C, low-density lipoprotein cholesterol; RBP4, retinol-binding protein 4;

* , *P*<0.05.

a Multivariate models were adjusted for age, gender, study area, physical activity, total calories, alcohol drinking and cigarette smoking. Non-drinkers were the reference group.

b Adjusted means reflect the estimated predictions when covariates were set as mean values.

c Overweight (BMI≥24 to <27 kg/m^2^) and obesity (BMI≥27 kg/m^2^) are determined according to the criteria defined by the Department of Health, Executive Yuan of Taiwan.

Table 3 shows the influence of the type of SSB consumed on TG and RBP4 levels. Drinkers of half-to-heavily HFCS-sweetened HSDs had multivariate and BMI-adjusted TG levels 19.46 mg/dl higher than non-drinkers. Adolescents who consumed HFCS-sweetened HSDs (11.96 to 12.33 ng/ml increases) and mixed sugar-sweetened BSDs (12.59 ng/ml increase) had increased RBP4 levels after accounting for the effects of BMI and UA.

**Table 3:** Multivariate-adjusted differences in triglyceride and retinol-binding protein 4 (RBP4) associated with the type of sugar-sweetened beverage consumed by adolescents.

Factors	Model I^a^		Model II^b^		Model III^b^	
	aDiff.	(95% CI)	aDiff.	(95% CI)	aDiff.	(95% CI)
**Triglyceride (mg/dl)**						
Non-intake	**Ref.**		**Ref.**		**Ref.**	
HFCS HSD (sweetened)^c^						
Slightly	13.51	(−8.07, 35.08)	13.02	(−8.23, 34.26)	9.75	(−11.10, 30.59)
Half-to-heavily	19.19*	(2.51, 35.88)	19.46*	(3.03, 35.90)	14.88	(−1.42, 31.17)
Mixed sugar BSD^c^	2.34	(−15.07, 19.75)	0.76	(−16.43, 17.95)	−4.35	(−21.42, 12.73)
**RBP4 (ng/ml)**						
Non-intake	**Ref.**		**Ref.**		**Ref.**	
HFCS HSD (sweetened)^c^						
Slightly	12.49*	(8.62, 16.36)	12.34*	(8.66, 16.03)	11.96*	(8.29, 15.63)
Half-to-heavily	12.79*	(9.79, 15.78)	12.87*	(10.02, 15.72)	12.33*	(9.46, 15.20)
Mixed-sugar BSD^c^	13.67*	(10.54, 16.79)	13.19*	(10.21, 16.18)	12.59*	(9.58, 15.60)

**Abbreviations**: aDiff., adjusted difference; HFCS, high-fructose corn syrup; HSD, hand-shaken sugar-containing drink; BSD, bottled sugar-containing drink;

* , *P*<0.05.

a Model I was adjusted for age, gender, study area, physical activity, total calories, alcohol drinking and cigarette smoking.

b Model II: Model I was additionally adjusted for body mass index. Model III: Model II was additionally adjusted for uric acid.

c HSD is only sweetened with HFCS, and BSD is sweetened with sucrose and/or HFCS.

The combined effects of the type and the amount of SSBs consumed on metabolic outcomes are shown in Table 4. Although all SSB drinkers showed higher TG levels, only drinkers with a daily intake of >500 ml half-to-heavy HSDs showed a significant increase (22.7 mg/dl). BMI accounted for 5.1% of the effect in this group of SSB consumers (Model IIA) and UA explained 32.6% of the effect (Model IIB). Allowing for the covariates considered, a 13.92 and 18.52 ng/ml higher RBP4 level was identified among adolescents who drank >500 ml/day of half-to-heavily HFCS-sweetened HSDs and mixed sugar-sweetened BSDs than non-drinkers, respectively. UA affected RBP4 more than BMI among high-quantity (>500 ml) HFCS-rich (half-to-heavy) HSD drinkers and BSD drinkers (2.4% and 3.0−3.7% of effect changes for BMI in Model IIA, and 9.0% and 7.9−8.9% of effect changes for UA in Model IIB, were respectively detected among the two groups of SSB drinkers).

**Table 4:** Multivariate-adjusted differences in triglyceride and retinol-binding protein 4 (RBP4) associated with the type and amount of sugar-sweetened beverage consumed by adolescents.

Factors	Model I^a^		Model IIA^b^			Model IIB^b^		
	aDiff.	(95% CI)	aDiff.	(95% CI)	EC^c^	aDiff.	(95% CI)	EC^c^
**Triglyceride (mg/dl)**								
Non-intake	**Ref.**		**Ref.**			**Ref.**		
HFCS HSD/Daily intake^d^								
Slight/1–500 ml	14.84	(−12.69, 42.37)	16.75	(−10.42, 43.93)		11.76	(−14.77, 38.29)	
Slight/>500 ml	12.49	(−14.66, 39.64)	9.67	(−17.18, 36.52)		7.62	(−18.61, 33.86)	
*P*_trend_ e	0.298		0.392			0.701		
Half-to-heavy/1–500 ml	13.64	(−7.08, 34.36)	16.08	(−4.43, 36.60)		13.19	(−6.74, 33.13)	
Half-to-heavy/>500 ml	22.70*	(4.38, 41.03)	21.55*	(3.47, 39.64)	−5.1%	15.29	(−2.71, 33.30)	−32.6%
*P*_trend_ e	0.041*		0.054			0.210		
Mixed-sugar BSD/Daily intake^d^								
BSD/1–500 ml	0.55	(−18.32, 19.41)	−0.92	(−19.55, 17.72)		−5.47	(−23.87,12.92)	
BSD/>500 ml	6.44	(−17.20, 30.08)	4.57	(−18.77, 27.92)		−2.21	(−25.35, 20.93)	
*P*_trend_ e	0.424		0.686			0.907		
**RBP4 (ng/ml)**								
Non-intake	**Ref.**		**Ref.**			**Ref.**		
HFCS HSD/Daily intake^d^								
Slight/1–500 ml	12.71*	(7.94, 17.49)	13.28*	(8.72, 17.83)	4.4%	12.19*	(7.58, 16.80)	−4.1%
Slight/>500 ml	12.56*	(7.86, 17.27)	11.73*	(7.23, 16.23)	−6.6%	11.74*	(7.18, 16.30)	−6.6%
*P*_trend_ e	<0.001*		<0.001*			<0.001*		
Half-to-heavy/1–500 ml	11.29*	(7.69, 14.88)	12.01*	(8.57, 15.45)	6.4%	11.21*	(7.75, 14.68)	−0.7%
Half-to-heavy/>500 ml	13.92*	(10.75, 17.10)	13.58*	(10.55, 16.61)	−2.4%	12.67*	(9.54, 15.79)	−9.0%
*P*_trend_ e	<0.001*		<0.001*			<0.001*		
Mixed-sugar BSD/Daily intake^d^								
BSD/1–500 ml	11.52*	(8.25, 14.79)	11.09*	(7.97, 14.21)	−3.7%	10.50*	(7.30, 13.70)	−8.9%
BSD/>500 ml	18.52*	(14.42, 22.62)	17.97*	(14.06, 21.88)	−3.0%	17.05*	(13.03, 21.07)	−7.9%
*P*_trend_ e	<0.001*		<0.001*			<0.001*		

**Abbreviations**: aDiff., adjusted difference; HFCS, high-fructose corn syrup; HSD, hand-shaken sugar-containing drink; BSD, bottled sugar-containing drink;

* , *P*<0.05.

a Model I was adjusted for age, gender, study area, physical activity, total calories, alcohol drinking and cigarette smoking.

b Model IIA: Model I was additionally adjusted for body mass index. Model IIB: Model I was additionally adjusted for uric acid.

c Effect change (EC) associated with the inclusion of an additional covariate into model I.

d HSD is only sweetened with HFCS, and BSD is sweetened with sucrose and/or HFCS.

e *P* values for dose-response trends were obtained based on the groups of non-drinkers, 1–500 ml/day and >500 ml/day drinkers.

**Figure 1:**
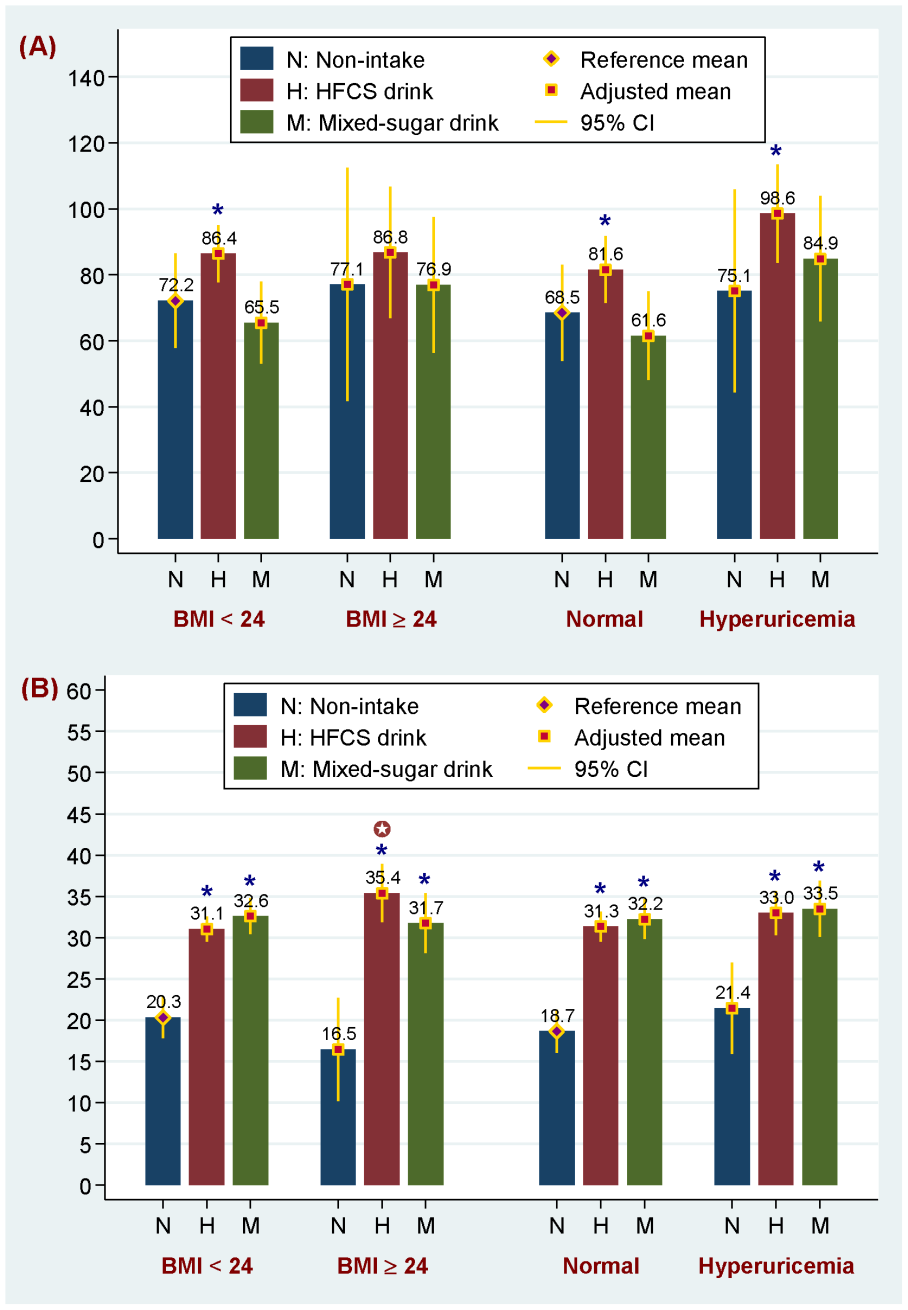
Interactive effects of the type of sugar-sweetened beverage (SSB) consumed, overweight/obesity and hyperuricemia on: (A) serum triglyceride (TG) and (B) retinol-binding protein 4 (RBP4) levels. **Note:** SSB groups were classified as non-intake, high-fructose corn syrup-containing drinks (HFCS drink) and sucrose/HFCS-mixed sugar-containing drinks (Mixed-sugar drink). Overweight (BMI≥24 to <27 kg/m^2^) and obesity (BMI≥27 kg/m^2^) are determined according to the criteria defined by the Department of Health, Executive Yuan of Taiwan. The multivariate-adjusted means were obtained using a linear regression model adjusted for age, gender, study area, physical activity, total calories, alcohol drinking and cigarette smoking, and where appropriate, the levels of serum uric acid and body mass index (BMI). * denotes a significant adjusted-mean difference as compared to the reference mean (*P*<0.05). ??? denotes a significant interaction effect on RBP4 levels that was detected among adolescents with overweight/obesity and consuming HFCS drinks (*P* for interaction = 0.037). The waist circumference was 67.7 and 68.0 cm for non-drinkers, 66.4 and 83.8 cm for HFCS drinkers, and 90.4 and 85.3 cm for mixed-sugar drinkers with a BMI<24 and ≥24 kg/m^2^, respectively.

Figure 1 shows the interactive effects of SSB consumption, overweight/obesity and hyperuricemia on serum TG and RBP4 levels. Compared with non-drinkers (68.5 mg/dl), HFCS-sweetened HSD drinkers with normal UA levels (81.6 mg/dl) and hyperuricemia (98.6 mg/dl) exhibited higher TG concentrations, and the TG circulating level was significantly higher in HSD drinkers with hyperuricemia than without (*P*<0.05). In adolescents with a BMI<24 kg/m^2^, consumption of HFCS-sweetened HSDs was associated with a 10.8 ng/ml significant increase in RBP4 levels as compared to non-drinkers, and in adolescents with a BMI≥24 kg/m^2^, the increase was interactively boosted to 15.1 ng/ml (*P* for interaction = 0.037). The effect of hyperuricemia on the increase in serum RBP4 levels in relation to the two types of SSB intake was limited. A comparable effect was observed among adolescents with and without hyperuricemia with an adjusted mean of 33.0 to 33.5 ng/ml and 31.3 to 32.2 ng/ml, respectively. The waist circumference was 67.7 and 68.0 cm for non-drinkers, 66.4 and 83.8 cm for HFCS drinkers, and 90.4 and 85.3 cm for mixed-sugar drinkers with a BMI<24 and ≥24 kg/m^2^, respectively.

The correlation of serum RBP4 levels with anthropometric and metabolic outcomes in different SSB consumption groups is shown in Table 5. In SSB non-drinkers, no parameters were linearly related to RBP4 levels. However, in HSD drinkers, RBP4 levels were correlated with waist circumference, body fat, BMI, UA and TG (correlation coefficient *r*, 0.253 to 0.404, *P*<0.05). By contrast, only the linear relationship between UA and RBP4 levels was noteworthy in the BSD group (*r* = 0.307).

**Table 5:** Correlations of retinol-binding protein 4 (ng/ml) with anthropometric and metabolic outcomes associated with the type of sugar-sweetened beverage consumed by adolescents.

Factors	Non-intake		HFCS HSD^a^		Mixed-sugar BSD^a^	
	(n = 40)		(n = 101)		(n = 59)	
	*r*	*P*	*r*	*P*	*r*	*P*
Waist circumference (cm)	0.117	0.471	0.373*	<0.001	0.210	0.111
Body fat (%)	0.195	0.228	0.359*	<0.001	0.121	0.363
Body mass index (kg/m^2^)	0.102	0.531	0.404*	<0.001	0.227	0.084
Uric acid (mg/dl)	0.280	0.081	0.253*	0.011	0.307*	0.018
Triglyceride (mg/dl)	0.247	0.124	0.262*	0.008	0.190	0.151

**Abbreviations:** HFCS, high-fructose corn syrup; HSD, hand-shaken sugar-containing drink; BSD, bottled sugar-containing drink; *r*, correlation coefficient;

* , *P*<0.05.

a HSD is only sweetened with HFCS, and BSD is sweetened with sucrose and/or HFCS.

## Discussion

This study investigates SSB consumption in adolescents aged between 12 and 16 years in urban, suburban and rural areas of Southern Taiwan. The results demonstrate that the intake of SSB was related to an increase in serum TG and RBP4 levels. Consuming HFCS-rich HSDs was significantly associated with increased TG levels, and serum UA contributed to this effect. HFCS-sweetened and mixed sugar-sweetened beverage consumption was linked to a higher RBP4 level. Furthermore, consuming HFCS-rich beverages and BMI values interactively reinforced serum RBP levels among overweight/obese adolescents.

Hepatic glucose metabolism is restrained by cytosolic ATP and citrate levels when energy conditions are high. By contrast, hepatic fructose metabolism is unrelated to energy conditions, therefore the hepatic uptake of dietary fructose and the metabolism of fructose to DNL substrates are unrestricted [32]. Human studies accounting for energy-balanced feeding have demonstrated that prolonged fructose—but not glucose—ingestion increases hepatic fractional DNL and reduces postheparin lipoprotein lipase [20], [21]. For TG production in the liver, elevated fructose-induced DNL supplies endogenous fatty acids and increases the intrahepatic availability of fatty acids by limiting the entry of fatty acid into the mitochondria by producing malonyl-CoA [20]. Reducing plasma TG clearance because of decreased lipoprotein lipase levels may also contribute to fructose-induced hypertriglyceridemia [20], [33]. Recent dietary SSB trials have shown that consuming fructose for 6 to 8 weeks at 17% to 25% of energy requirements increases the postprandial and daily TG concentration more than consuming isocaloric glucose among adults [20], [34]. This study identified a higher BMI-adjusted fasting TG level among adolescents who consumed half-to-heavily sweetened HFCS beverages (19.46 mg/dl increase) than among those who consumed mixed sugar-sweetened beverages (0.76 mg/dl increase).

Monosaccharide fructose is the only carbohydrate that increases serum UA levels by activating the fructokinase pathway and degrading the purine nucleotide [35] and by upregulating de novo purine nucleotide synthesis in hepatocytes [36]. This study found that adolescents who consumed >500 ml/day of half-to-heavily sweetened HFCS beverages had a TG concentration 22.70 mg/dl higher than non-drinkers, and UA levels explained 32.6% of this increase. On the other hand, HFCS-rich HSD drinkers and mixed sugar BSD drinkers with hyperuricemia both had a noticeably higher TG level than those drinkers with normal UA levels (Figure 1, 98.6 vs. 81.6 mg/dl in HFCS-rich drinkers, and 84.9 vs. 61.6 mg/dl in mixed sugar drinkers, both *P*<0.05). A significant positive correlation between UA and TG levels was also identified among HSD drinkers (*r* = 0.295, *P* = 0.003) and among BSD drinkers (*r* = 0.360, *P* = 0.005), but not among non-drinkers (*r* = 0.274, *P* = 0.087, data not shown). These findings demonstrated an association between circulating UA and TG levels among adolescents who consumed SSB beverages (HFCS-rich HSD and mixed sugar BSD beverages). A recent biomedical experiment demonstrated that UA up-regulates fructokinase expression and augments the lipogenic effects induced by fructose in human hepatocytes [37]. It also showed that constraining UA production substantially restrains the accumulation of fructose-induced TG [37]. Studies have suggested that fructose-induced hyperuricemia may mediate certain abnormalities related to cardiometabolic risk factors, such as hypertriglyceridemia, hypertension and insulin resistance [21], [38].

Transgenic animal experiments have demonstrated that reduced expression of adipose-specific GLUT4 increases circulating RBP4 levels in adipose tissue, and that increased RBP4 expression promotes hepatic glucose production by activating phosphoenolpyruvate carboxykinase and impairs insulin signaling in muscle [3], [5]. Increased serum RBP4 levels in humans are associated with obesity, metabolic syndrome, type 2 diabetes and cardiovascular disease [3], [6]–[9]. Furthermore, decreased plasma RBP4 levels have been identified in patients with specific medical interventions, such as diet, exercise, oral antidiabetic drugs and hypolipidemic agents [3], [9]. A study of adults linked prolonged dietary intake of fructose to higher serum RBP4 levels [21]. Using three SSB consumer groups (non-drinkers, 1−500 ml/day and >500 ml/day drinkers) for evaluation, this study demonstrates that a dose-response relationship between SSB intake and circulating RBP4 levels exists in adolescents, regardless of the type of SSB consumed. This dietary factor is also a major contributor to several cardiorenal diseases [38]–[40].

A recent dietary SSB intervention study of overweight and obese participants showed that consuming fructose-sweetened beverages at 25% of energy requirements for 10 weeks promotes hepatic DNL and increases intra-abdominal fat accumulation [20]. In humans, RBP4 expression is associated with larger adipocytes, and visceral adipose tissue exhibits a higher RBP4 concentration than subcutaneous adipose tissue [41]. This study shows that the effect of HFCS-sweetened HSD consumption on increased serum RBP4 levels was significantly strengthened among overweight/obese adolescents than among normal weight adolescents (RBP4 = 35.4 ng/ml, compared to 31.1 ng/ml, *P* for interaction = 0.037, Figure 1). Biomedical studies have suggested that decreasing GLUT4 expression in adipose tissue and increasing TG deposition into visceral adipose tissue may be the mechanism through which fructose exerts these effects [20], [21]. This study shows that the waist circumferences of HFCS HSD drinkers with BMIs≥24 kg/m^2^ (90.4 cm) were considerably higher than those of non-drinkers with BMIs<24 kg/m^2^ (67.7 cm).

Our findings show that circulating RBP4 levels are significantly correlated with waist circumference, body fat, BMI and serum TG levels among HFCS-sweetened HSD drinkers, but not among mixed sugar-sweetened BSD drinkers and non-drinkers. In this study, the body-weight-associated parameter values of adolescents who consumed HFCS-rich beverages were higher than those of adolescents who consumed mixed-sugar beverages. HSD drinkers also had a higher circulating level of TG than did BSD drinkers. In epidemiological studies, increased serum RBP4 levels have been linked to obesity and increased serum TG levels [41], [42]. These findings partly explain why the relationships between RBP4 and body-weight-related variables and TG are evident in the HSD drinkers but not in the BSD drinkers. Our results support the previously proposed hypothesis that high fructose consumption has a significant effect on the association of RBP4 with obesity and TG. [20], [21]. Alternatively, similar to findings from patients with type 2 diabetes [43], [44], a significant linear relationship exists between serum RBP4 and UA levels among healthy adolescents who consumed HFCS-rich HSD beverages (*r* = 0.253) and mixed sugar BSD beverages (*r* = 0.307). RBP4 is a novel cardiometabolic risk factor [9], and fructose-derived hyperuricemia may be a critical intermediate agent for the abnormalities of cardiometabolic risk factor [21], [38]. These findings emphasize the importance of SSB consumption on the adverse effects of cardiometabolic disease.

The results of this study are subject to certain limitations. Although it is improbable that the reported SSB consumption was biased by participant knowledge of identified UA levels (because clinical blood examinations were conducted 3 weeks after data collection), the cross-sectional nature of this study precludes any causal interpretations. Because anthropometric and clinical outcomes were measured only once, this investigation only presents a snapshot of serum lipid profile and RBP4 in the study participants. The main strength of this investigation is that it is the first study to report the association between SSB intake and circulating RBP4 levels among healthy adolescents. Because serum RBP4 is also affected by a number of non-metabolic circumstances, such as acute illness, injury, liver and renal failure [9], respondents with these conditions were precluded from the study. Furthermore, all of the evaluations considered critical confounding factors.

In conclusion, high-quantity (>500 ml) HFCS-rich (half-to-heavy) beverage intake is associated with higher TG levels, and serum UA may be connected with this increase. Hyperuricemia is likely to intensify the effect of HFCS-rich SSB intake on elevated TG levels, and being overweight/obesity may modify the action of fructose on higher circulating levels of RBP4.
